# A modified density gradient proteomic-based method to analyze endolysosomal proteins in cardiac tissue

**DOI:** 10.1016/j.isci.2021.102949

**Published:** 2021-08-04

**Authors:** Thamali Ayagama, Samuel J. Bose, Rebecca A. Capel, David A. Priestman, Georgina Berridge, Roman Fischer, Antony Galione, Frances M. Platt, Holger Kramer, Rebecca A.B. Burton

**Affiliations:** 1 University of Oxford, Department of Pharmacology, Oxford, OX1 3QT UK; 2 Target Discovery Institute, University of Oxford, Oxford, OX3 7FZ UK; 3 Biological Mass Spectrometry and Proteomics Facility, MRC London Institute of Medical Sciences, Imperial College London, London, W12 0NN UK

**Keywords:** Porcine cardiology, Biological sciences, Biochemistry, Systems biology, Omics, Proteomics

## Abstract

The importance of lysosomes in cardiac physiology and pathology is well established, and evidence for roles in calcium signaling is emerging. We describe a label-free proteomics method suitable for small cardiac tissue biopsies based on density-separated fractionation, which allows study of endolysosomal (EL) proteins. Density gradient fractions corresponding to tissue lysate; sarcoplasmic reticulum (SR), mitochondria (Mito) (1.3 g/mL); and EL with negligible contamination from SR or Mito (1.04 g/mL) were analyzed using Western blot, enzyme activity assay, and liquid chromatography with tandem mass spectrometry (LC-MS/MS) analysis (adapted discontinuous Percoll and sucrose differential density gradient). Kyoto Encyclopedia of Genes and Genomes, Reactome, Panther, and Gene Ontology pathway analysis showed good coverage of RAB proteins and lysosomal cathepsins (including cardiac-specific cathepsin D) in the purified EL fraction. Significant EL proteins recovered included catalytic activity proteins. We thus present a comprehensive protocol and data set of guinea pig atrial EL organelle proteomics using techniques also applicable for non-cardiac tissue.

## Highlights

• Method to analyze endo-lysosomal proteins from guinea pig cardiac atrial tissue
• Endolysosome organelle proteomics using techniques applicable for non-cardiac tissue
• We identified endo-lysosomal marker proteins such as Rab7A, VPS29, RILP, and LAMTOR1-5

## Graphical abstract

**Figure:**
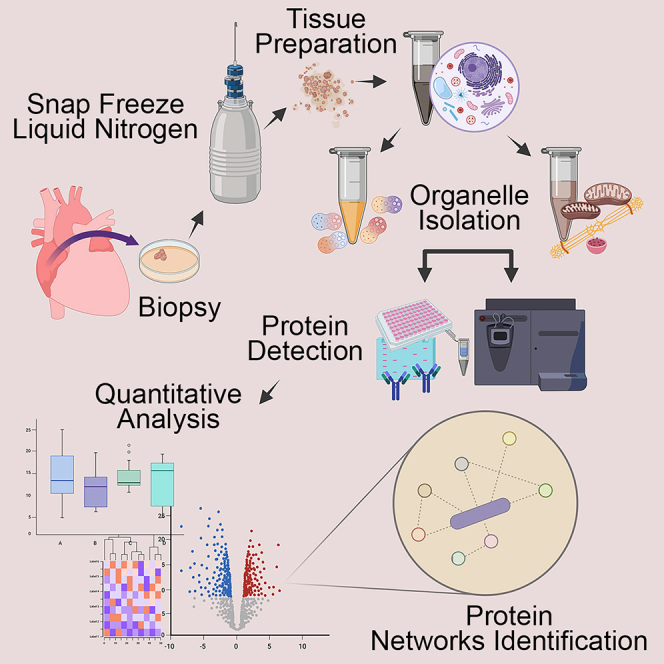

## Introduction

The concept of understanding proteins from a compartmentalization perspective, their interconnected properties, and dynamic distribution in health and disease is critical for deciphering the phenotype of a cell (Larance and Lamond, 2015). Significant advances in mass spectrometry-based proteomics allow scientists to achieve multidimensional measurements of proteins with greater efficiency, enabling for example the generation of more detailed maps of the human proteome (Kim et al., 2014). Relative quantification methods of samples include label-free quantification (Hoffert et al., 2006), *in vivo* metabolic stable isotope labeling (Wang et al., 2002), stable isotope labeling using chemical tags that are covalently attached *in vitro*, tandem mass tags, and isobaric tags for relative and absolute quantification (recently reviewed by Larance and Lamond (2015)).

Methods available to analyze subcellular protein localization in cells and tissues are diverse (Graham, 2001; Walker and Lloyd-Evans, 2015). Depending on the cell or tissue to be analyzed, the different methods have distinctive advantages and disadvantages. The subcellular fractionation methods most commonly combined with mass spectrometry-based analysis include differential centrifugation and either equilibrium gradient centrifugation or non-equilibrium gradient centrifugation (Larance and Lamond, 2015). One of the challenging issues encountered with subcellular fractionation is due to very small density differences between individual organelle fractions (Wolff and Pertoft, 1972; Hoglinger et al., 2019). Advanced methods, such as localization of organelle proteins by isotope tagging (LOPIT (Dunkley et al., 2004)), offer advantages to differentiate large organelles, small intracellular vesicles, and even large complexes such as ribosomes, purely based on their density and not requiring isolation and purification of organelles. An increased understanding of the physiological and structural interactions between intracellular organelles, such as the role of membrane contact sites (MCSs) (Hoglinger et al., 2019) and inter-organelle nanojunctions in regulating physiological function (Fameli et al., 2014), also raises considerations for determining fraction purity. For example, Niemann-Pick type C protein 1 is now known to play a role in regulating MCSs between lysosomes and the endoplasmic reticulum (ER) (Hoglinger et al., 2019). Such interactions raise the possibility, for example, of contamination of ER fractions by lysosomal proteins as a result of MCS formation.

Over recent years, significant progress has been made in establishing a region and cell-type resolved quantitative proteomic map of the human heart (Doll et al., 2017). The value of such approaches has been demonstrated by the application of these data to define molecular changes in patients suffering from cardiovascular disease and to provide comparisons with known genomic parameters for cardiovascular disease including heart failure and atrial fibrillation (AF) (Kaab et al., 2004; Linscheid et al., 2020). Mishandling of Ca^2+^ regulation in cardiac cells is closely linked to the pathophysiology of cardiac arrhythmias such as AF (Capel et al., 2021), and there is increasing evidence for an involvement of lysosomes in cardiac Ca^2+^ handling/mishandling (Aston et al., 2017; Capel et al., 2015). In order to understand the contribution of the organelles involved in Ca^2+^ regulation (including lysosomes in addition to sarcoplasmic reticulum [SR] and mitochondria [Mito]) of atrial function, a more detailed organelle-specific approach is required. The guinea pig (*Cavia porcellus*) is a common and well appreciated small mammal model used in cardiovascular research, and recent work from our own group has highlighted the value of using *C. porcellus* tissue to study Ca^2+^ handling in atrial cells (Capel et al., 2021; Capel and Terrar, 2015). *C. porcellus* cardiomyocyte electrophysiology, which includes the typical long plateau phase of the action potential, is closer to that of human compared with mouse or rat. Our proof-of-concept study offers the advantage of scalability, involving utilization of very small quantities of heart biopsies, for instance those obtainable during surgery. Using this approach, we will be able to explore the contribution of lysosomes in health and disease. In doing so, we hope to unravel mechanistic insights, relating to changes in protein composition and abundance in disease models that can then be related back to functional data.

The importance of lysosomes in cardiac physiology, both in health and in disease, has long been recognized (Wildenthal and Decker, 1980). Early elegant ultrastructural and biochemical studies investigated the levels of lysosomal enzyme activity in many organs including the heart (Comolli, 1971; Wilson, 1972; Youhotsky-Gore and Pathmanathan, 1968; Traurig, 1976). As early as 1964, an increased number of lysosomes were observed in the atrial muscle of chronically diseased or stressed hearts with acquired heart disease such as mitral stenosis (Wheat, 1965). In addition, Kottmeier et al. (Kottmeier and Wheat, 1967) conducted studies in a dog model of atrial septal defects, and their data demonstrated an increase in the number of myocardial lysosomes in cells subjected to increased metabolic demands. The correlation however between the degree of stress and elevation in lysosome count could not be determined from these early studies. In 1977, Wildenthal et al. (Lundholm and Scherstén, 1975; Wildenthal et al., 1977) looked at differences in cardiac lysosomal enzymes in detail and confirmed previous observations (Comolli, 1971; Hendley and Strehler, 1965) correlating increased age with the total activity of the lysosomal proteinase, cathepsin D, further highlighting links between lysosomal function and cardiac disease.

Development of techniques that facilitate proteomic characterization of individual organelles (Au et al., 2007) could provide valuable information regarding the function of lysosomal pathways in normal and disease states (Capel et al., 2015). For instance, lysosomal calcium signaling via the nicotinic acid adenine dinucleotide phosphate (NAADP) pathway (Bak et al., 2001; Galione et al., 2010).

Relatively little is known about the protein composition of the lysosomes in cardiac atria. In this study, we developed a flexible, low-cost, modified density gradient method for endolysosomal (EL) organelle isolation, allowing better organelle protein identification from the processing of small amounts of frozen cardiac atrial tissue biopsies. We performed label-free, quantitative mass spectrometry that allows us to better appreciate lysosomal function in physiological and pathophysiological states. Furthermore, Western blot analysis and lysosomal enzymatic assays showed that the protein content and enzymatic activity of the EL fraction were as expected, with minimum contamination from other organelles. Organelle-specific quantitative proteomics approaches such as this can help progress our understanding of the role of lysosomes in atrial physiology and pathophysiology, for example by comparing protein composition from disease tissue samples or cell lines with those of healthy donors or patients.

Creation of an atrial EL organelle database offers valuable data in studying cardiac physiology. Using this method, we identified EL marker proteins (D'Souza et al., 2019) such as Rab7A, VPS29, MAN2B1, LAMTOR1, LAMTOR2, LAMTOR3, LAMTOR5, RILP, ACP2, GBA, and GAA in our proteomics data, as well as LAMP2 from Western blot data. Furthermore, statistical analysis by volcano plot of quantified protein hits revealed 564 EL proteins significantly enriched in the EL fraction (false discovery rate of 0.05).

## Results

### Density gradient approach toward acidic organelle isolation from *C. porcellus* atria

**Figure 1:**
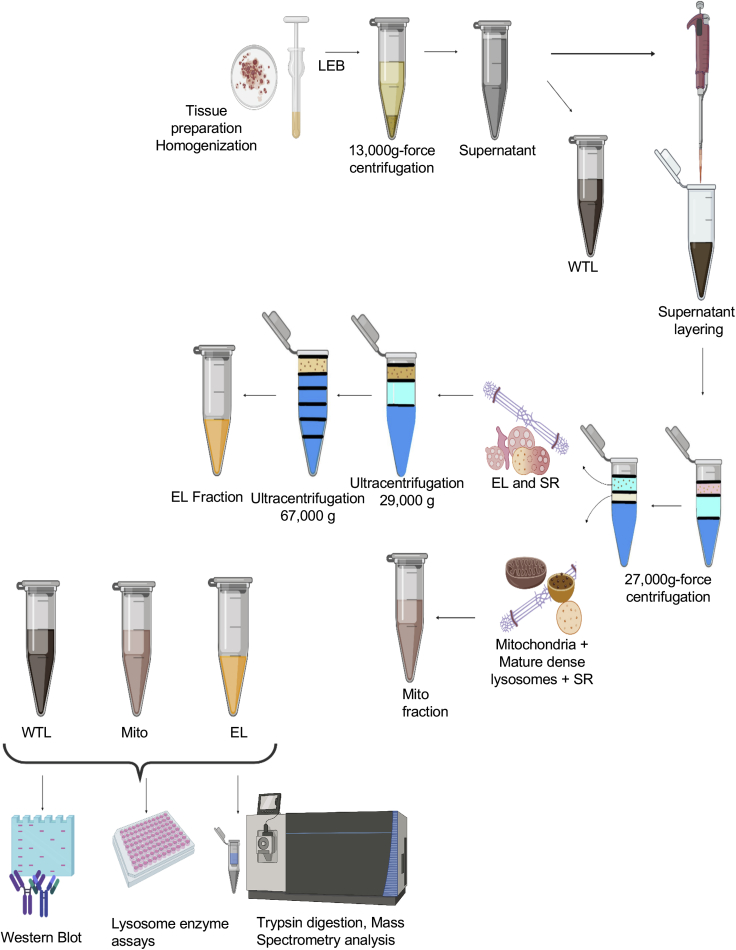
Flow chart of the acidic organelle isolation Top: Atrial tissue is homogenized using a Dounce homogenizer; after adding Lysosome enrichment buffer (LEB), tissue lysate is briefly centrifuged at 13,000 g-force, 4°C for 2 min; supernatant is collected without disturbing the tissue pellet (tissue lysate, TL); centrifuge tube is layered with 750 μL of 2.5 M sucrose, 250 μL of Percoll, and 200 μL of TL; centrifuge at 27,000 g-force, 10°C for 50 min: The mitochondria and SR accumulate at the biface of Percoll and 2.5 M sucrose and are carefully collected (mitochondria + SR enriched fraction). The area above the turbid white layer consists of endosomes and endolysosomes with minimum contamination of SR, and this fraction is collected for further removal of SR by centrifuging at 29,000 g-force 15°C for 30 min; for the differential density gradient centrifugation step, centrifuge tube is carefully loaded with the different density gradients made of sucrose, Percoll and ddH_2_O (starting from 1.3 g/mL, 1.11 g/mL, 1.07 g/mL, 1.05 g/mL, 1.04 g/mL). The fraction collected from above the turbid white layer is pipetted (200 μL) on top of the 1.04 g/mL Percoll; the tube is centrifuged at 67,000 g-force at 4°C for 30 min, and the top fraction consists of endolysosomes + endosomes. Bottom: Protein validation is performed using Western blot, lysosome enzyme assay, and proteomic analysis. (Flow chart created using Biorender.com).

An overview of the workflow is shown in Figure 1. In order to capture lysosomal-specific proteome data in guinea pig (*C. porcellus)* atrial frozen biopsies, we further developed a density gradient organelle isolation protocol based on previous work (Graham, 2001; Lamberti et al., 2015). The first stage was to eliminate tissue debris and plasma membrane by brief ultra-centrifugation. The supernatant enriched in SR, Mito, lysosomes, endosomes, and endolysosomes (ELs) were then separated by differential density gradient-based ultra-centrifugation. The much denser organelles such as crude Mito with mature lysosomes and most of the SR content were separated from the soluble fraction at this stage. A high degree of purity of the EL-enriched fraction was achieved by a repeated ultra-centrifugation step and confirmed by Western blot (Figure S1). The final fraction was then subjected to differential density gradient levels to partition proteins into specific compartments with respective buoyant densities.

### Validation of lysosomal proteins using lysosomal enzymes and immunoblotting assays

**Figure 2:**
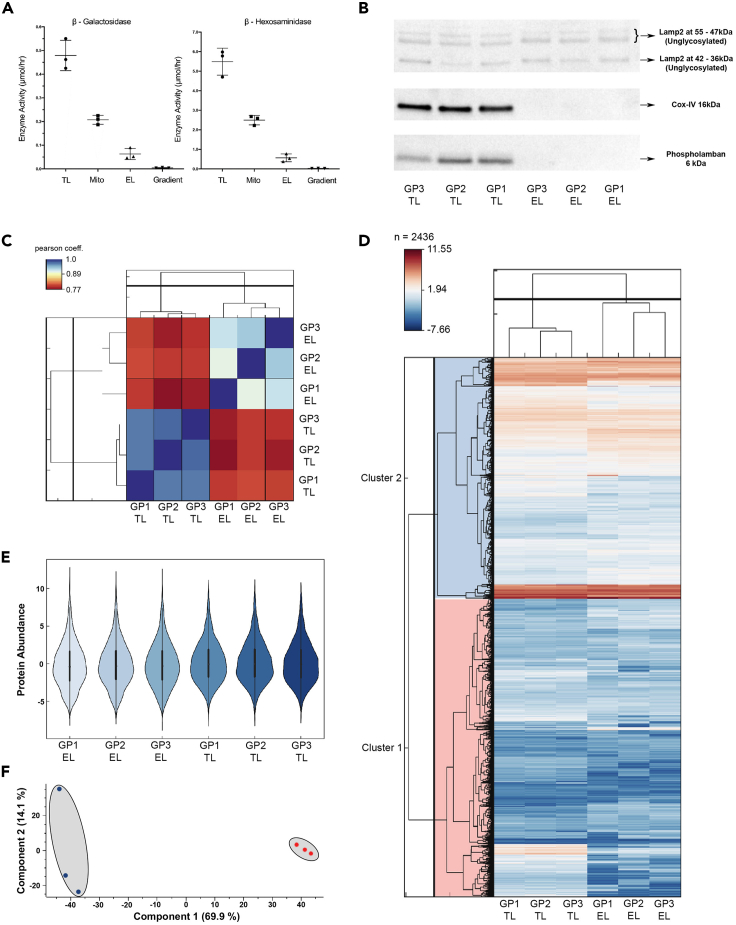
Expression of protein abundancy and technical reproducibility (A) Beta – galactosidase and Beta – hexosaminidase enzyme activities in adult guinea pig atria (n = 3). Lysosomal hydrolase activities (total Units ± SD) were measured in EL, Mito (which contain dense lysosomes) and as well as TL using artificial 4-MU-substrates. (B) Western blots performed in guinea-pig atrial tissue: LAMP2, COXIV and Phospholamban (n = 3). Identification of lysosome, mitochondria, and SR organelle levels between (TL) and EL fraction. For clarity, only relevant rows and columns are shown. The complete blot is provided in Figure S2A. (C) Pearson co-efficient correlation plot values show the positive or direct correlation between the reliability of the triplicated samples. (D) Heatmap of z-scored protein abundances (LFQ intensities) of the differentially expressed proteins after unsupervised hierarchical clustering. (E) Violin plot shows distribution of peptide abundance from EL fraction to TL among the triplicates. (F) Principal component analysis (PCA) of the six atrial samples based on their proteomic expression profiles. Each data point represents the total protein groups in each sample as a single vector. The components 1 and 2 represent the spatial resolution among the vectors. The average of vectors corresponds to a point in the K-space. Component one explains 69.9% of the variation, component two 14.1%. Red: TL, Blue: EL. Panels (C and D) generated using Perseus 1.5.2.4 and redrawn using Instant Clue (Nolte et al., 2018).

The main fractions, tissue lysate (TL), EL, and Mito, were validated for organelle enrichment by lysosome enzyme assays and Western blotting (Figures 2A and 2B). Lysosomal activities of β-galactosidase and β-hexosaminidase were assayed in TL, Mito, EL, and the remaining gradient fractions from Dunkin Hartley guinea pig atria (Figure 2A, N = 3). Figure 2A shows total units of activity for each enzyme in each fraction. Volumes, protein amounts, and specific activities for the enzymes are shown in Data S1.

Western blotting was conducted to analyze the presence of EL membrane proteins by blotting against LAMP2 (Figure 2B). The absence of the predominant organelles such as SR and mitochondrial membranes were examined by the SR marker protein phospholamban and inner mitochondrial membrane marker protein COX IV. We observed clear band visibility of LAMP2 in all the biological replicates of EL and TL. Phospholamban and COX IV were negligible in EL (Figures 2B and S2). In addition, Western blotting was carried out using glyceraldehyde 3-phosphate dehydrogenase as a loading control (Figure S2B).

Sample intensity distributions showed high similarity, with Pearson correlation coefficients of >0.9 (TL) and >0.8 (EL) for intra-group comparisons (Figure 2C), with density histograms of the LFQ intensity data assuring the near-normal distribution of protein intensities between the TL and EL in three biological replicates showing technical reproducibility (Figure S3A). In comparison, the distribution of protein intensities from our data was compared with that previously published for human atria by Doll et al. (2017), with both data sets showing similar patterns of distribution (Figure S4).

### Differential protein distribution using quantitative proteomic analysis

For overall assessment of functional protein resemblance between the fractions, unsupervised hierarchal clustering was employed on 2436 *C. porcellus* proteins. Gene ontology annotations identified statistically different abundance of protein groups between the fractions (FDR<0.05). A heat map was generated (Figure 2D), where color representation from blue to red demonstrates lowest to highest relative abundance of the protein groups in EL compared with TL. The protein groups represented in white showed no significant difference between the protein intensity leading to different levels of expression in the protein groups. The differential distributions of protein abundance from EL to TL are demonstrated using violin plots in Figure 2E. Principal component analysis (PCA) reduced the data dimensions for simpler interpretation (Figure 2F). As indicated in Figure 2F, vector deviation of 69.9% was observed between TL (red symbols) and EL fractions (blue symbols). An exceptional 14.1% segregation was observed in first biological replicate of EL. We observed higher level of mitochondrial proteins in the TL vectors than in EL, such as proteins involved in electron transport chain (eg: A0A286XXR8/NDUFB4, H0V9U7/NDUFB9 (Wu et al., 2016)) (Data S2), whereas EL contained proteins from endosomal and endocytic trafficking pathways (e.g.: A0A286XGA7/TOM1 (Masters et al., 2017), A0A286XNV7/ADIPOQ (Wang et al., 2017), A0A286Y1Z7/CDC42 (Sirokmány et al., 2006)) (Data S3). The molecular functional differences were clearly distinguished in the PCA plot.

### Functional networks within endolysosomes

**Figure 3:**
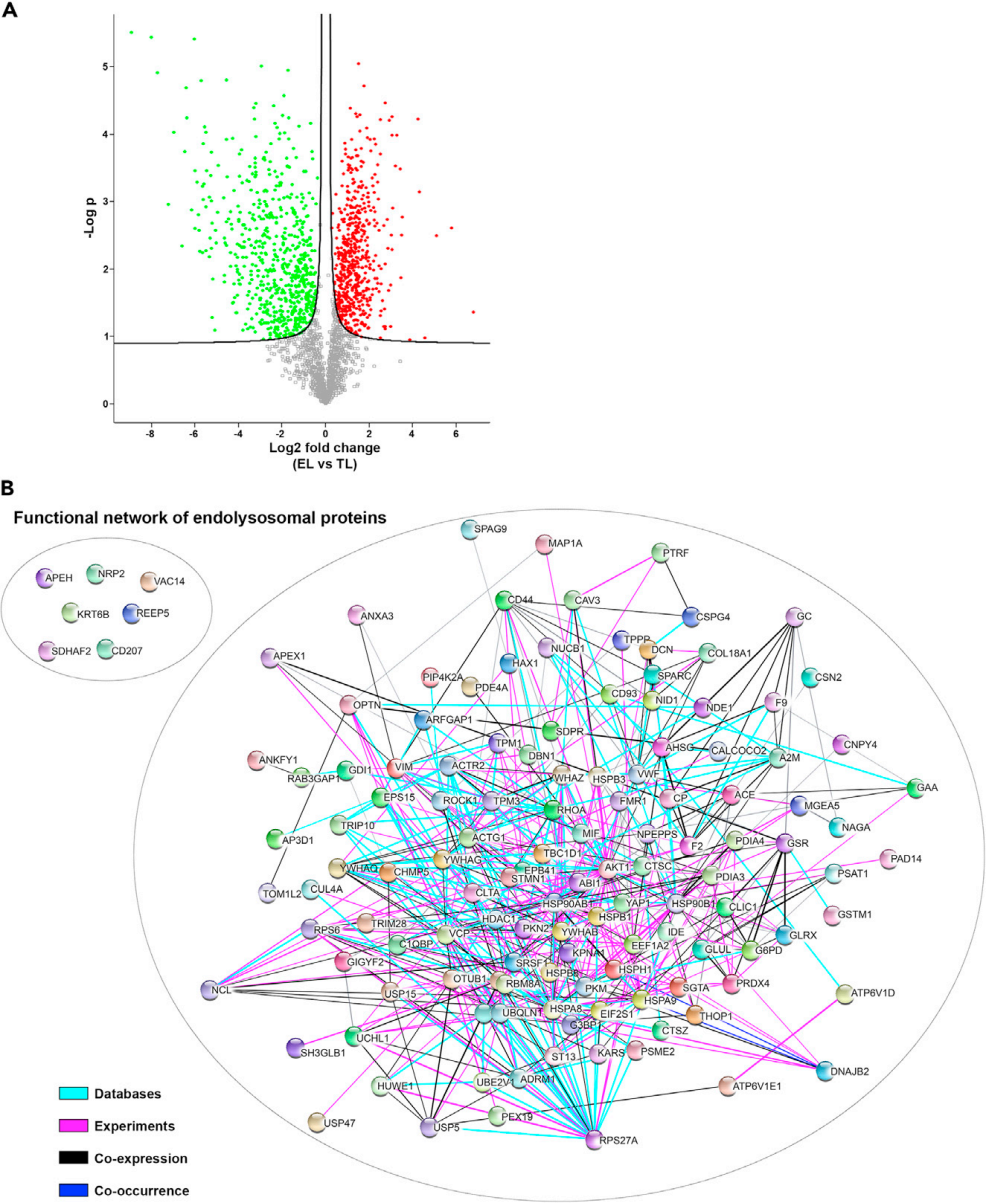
Profile of the endolysosomal proteins in EL fraction at a glance (A) Volcano plot is plotted against the –log2 transformation of the p values vs. the protein abundance differences in EL and TL. Significantly higher abundant proteins in EL compared with TL are highlighted in red and less abundant in green, respectively (FDR 0.05). Complete lists of proteins shown in red or green are provided in Data S2 and S3. (B) EL fraction proteins were clustered using Cytoscape consortium 3.7.2 with String, KeGG, GO, and Reactome pathway annotations. Proteins were clustered using median confidence score (0.4) and the molecular pathway parameters (edges) were filtered to databases, experiments, co-expression, and co-occurrence.

Proteins demonstrating differential abundance between TL and EL fractions were identified using volcano plot (Figure 3A). Of a total of 2,436 quantified proteins, 690 accounted as depleted in EL, demonstrating higher abundance in TL fractions (Figure 3A, green and Data S2), whereas 564 proteins accounted for the most enriched hits corresponding to higher abundance in EL (Figure 3A, red and Data S3). The functional networks of the quantified, statistically significant enriched proteins acquired from volcano plot analysis were mapped using several genomic and proteomic annotations (detailed description in methods section). The STRING network created by Cytoscape generated 125 proteins involved in a single functional network, and 7 proteins displayed a detached cluster that were not connected to any functional edge (Figure 3B and Data S4 and S5).

The biological interactions of the leading network (Figure 3B) display a single network which is based on 4 functional annotations. These annotations are based on information from published databases, experiments, co-occurrence and co-expression data (www.cytoscape.org). A color scale depicts the functional annotations, and the fading of the color displays the strength of the evidence. In addition to the EL network analysis, we performed a separate functional enrichment analysis using gene ontology (GO) pathway analysis; the most abundant protein IDs of the EL fraction were converted to human protein IDs and uploaded to the GO analysis software ShinyGO v0.61 (Ge et al., 2020). Using cell component filtration, the 10 most significant cell components were identified. The highest number of protein IDs (188) belonged to the vesicle trafficking component, suggesting the identified EL proteins are localized to the endolysosome route of the cell (Schroder et al., 2007; Martin-Serrano et al., 2003) (Data S6).

### PANTHER gene annotation pathway analysis

**Figure 4:**
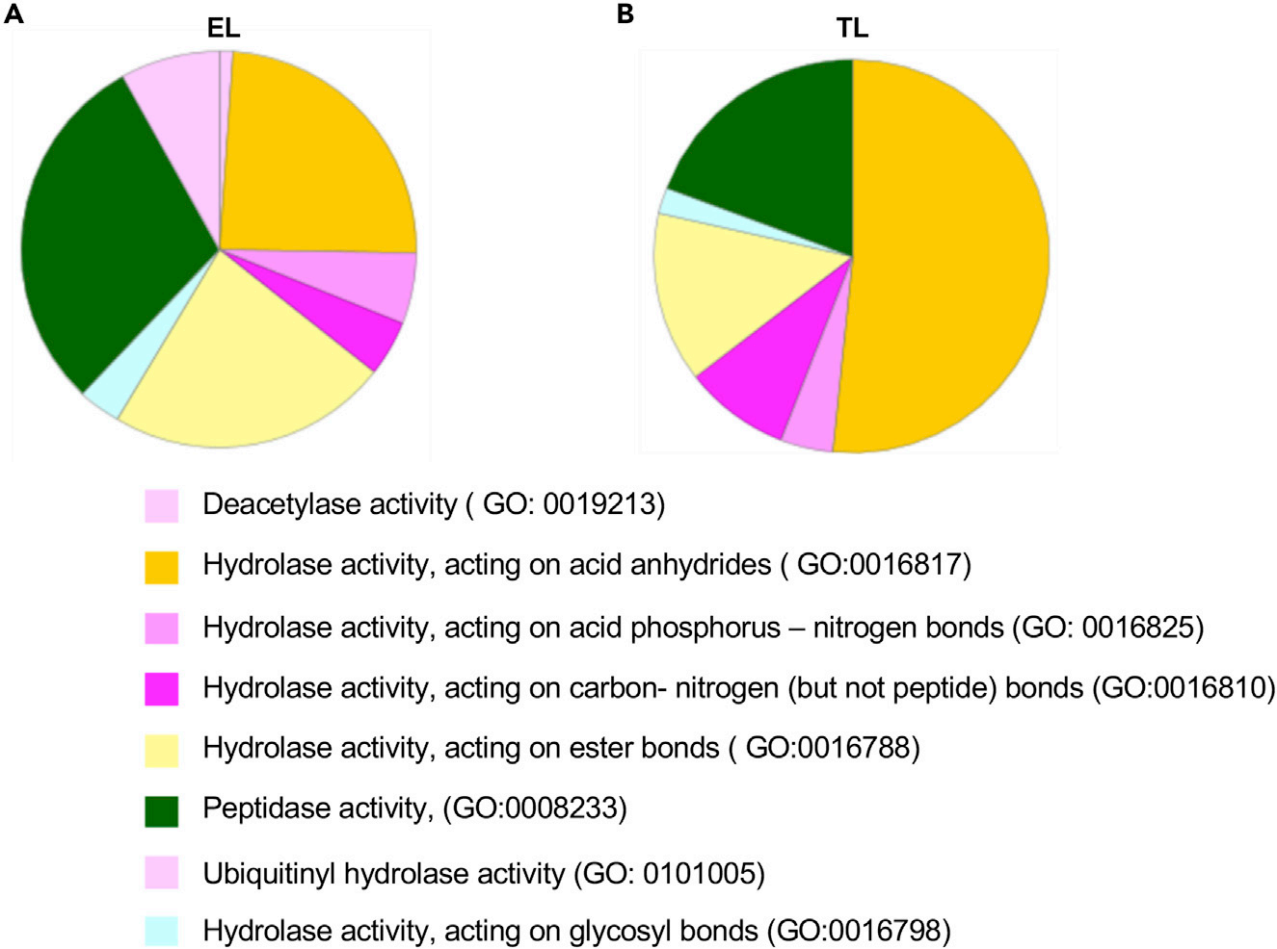
Gene ontology panther pathway analysis (A and B) The molecular function of the endolysosome fraction (EL) showed 39% of catalytic activity, whereas the molecular function of the tissue lysate (TL) showed 42.8% of catalytic activity. The catalytic hydrolase activity was further analyzed for individual hydrolase activity, EL fraction (A) showed higher peptidase activity compared with TL (B).

The protein profile of EL (Figure 4A) and TL (Figure 4B) was analyzed using the PANTHER pathway database (www.pantherdb.org) using a total of 1,254 quantified proteins (Data S6). The most enriched EL fraction proteins (564) and the proteins most abundant in TL (690) were plotted with mapped gene identifiers, using pie charts to demonstrate the overall representation of protein groups by molecular function categories. The EL fraction and TL displayed, respectively, 39% and 42.8% of the catalytic activity. The catalytic representation of the EL and TL was categorized according to the hydrolase enzyme groups. The percentages between EL and TL were, respectively, as follows: peptidase (29.9%, 19.4%), hydrolase acting on carbon-nitrogen (but not peptide) (4.8%, 8.6%), hydrolase acting on ester bonds (23.8%, 14%), hydrolase acting on acid anhydrides (25%, 51.6%), hydrolase activity acting on glycosyl bonds (2.4%, 2.2%), and hydrolase activity acting on acid phosphorus-nitrogen bonds (4.8%, 4.3%). Lysosome-specific cathepsins were highly abundant in the peptidase category found in the EL fraction. In contrast, cytochrome oxidases of mitochondrial origin were highly abundant in TL.

### Organelle expression profile

Our data demonstrate the presence of lysosomal markers β-galactosidase, β-hexosaminidase, Rab7A, and LAMP2 in our EL preparations and the absence of markers from mitochondria (COX IV) and ER (Calnexin protein) (Bagshaw et al., 2005), with fewer cytochrome C reductase proteins (ER marker; H0VNA2/CYC1, A0A286Y030/NDUFA4 in EL and A0A286XMD6/UQCRFS1, CYC1, NDUFA4, A0A286XTF9/UQCRB, H0W408/UQCRQ, H0VIM6/UQCR10 in TL) (Bagshaw et al., 2003). Key proteins identified are summarized in Table S1.

### Pathway analysis of EL, endosome, and lysosomal proteins

The majority of the proteins identified had a direct connection with the structural biology of endolysosomes, lysosomes, and functions related to their membranes, whereas some proteins identified had a functional role in lysosome biogenesis and endocytic processes. The following sections provide details of some of the key proteins identified in our study, as well as their known associations according to previously published data.

#### a) Proteins involved in endosomal/lysosomal functions

We identified multiple proteins linked to endocytosis, including AP2A1, LRP1, and APOE (Actis Dato et al., 2020). AP2A1 is a key regulator of endosomal/lysosomal protein sorting pathways (Nakatsu and Ohno, 2003), whereas LRP1 and APOE are involved in cholesterol metabolism (Bilousova et al., 2019). H0UWL7/ACE and H0VDM6/ITGB1 have been recognized in hypertrophic cardiomyopathy disease pathways (Yuan et al., 2017; Wang et al., 2020). HSPA8 is linked to Parkinson's disease, where it is involved in the impairment of lysosomal autophagy. HSPA8 may contribute to lysosomal storage disorders (LSDs) as a functional component of lysosome vesicle biogenesis (Rudenok et al., 2019). A large number of Rab proteins were categorized under Rab-regulated trafficking, membrane trafficking, and endocytosis (Table S1). We found numerous proteins, such as V-type ATPase proton pumps commonly expressed in lysosomes and lysosome-related organelles, including ATP6V1D (A0A024R683) (Schroder et al., 2007) (Table S1). In addition, we identified the endosomal proteins Flotillin-1 (A0A286XE27) (Schroder et al., 2007) and CHMP5 (Q9NZZ3) (Martin-Serrano et al., 2003) in our EL fractions.

Atrial tissue-specific protein markers such as myosin heavy chain 6 (MYH6/A0A286Y2B6), myosin heavy chain 7 (MYH7B/H0V2M2), peptidylglycine alpha amidating monooxygenase (PAM/H0VJZ4), and natriuretic peptide A (NPPA/H0VXX0) reported in previous cardiac proteomic studies (Doll et al., 2017) were identified in our TL (Table S1).

#### b) Acidic organelle proteins related to cellular Ca^2+^ and ion channels

EEA1, a phosphoinositide binding domain, and β1-integrin, which has a functional role related to two-pore channels (TPCs), were observed in our proteomic profiles (Nguyen et al., 2017; Itoh and Takenawa, 2002). Inhibition of TPC function in metastatic cancer cells has been shown to prevent trafficking of β1-integrin, leading to accumulation within EEA1-positive early endosomes and preventing cancer cell migration (Nguyen et al., 2017).

EH domain-containing proteins function as retrograde transport regulators and retrograde trafficking mediates the transport of endocytic membranes from endosomes to the *trans*-Golgi network (Zhang et al., 2012). Our study identified several proteins involved in retrograde transport and trafficking including EHD 2 and 4, Ankyrin, Vps 35, and Annexin A. A 2010 study by Gudmundsson et al. (2010) showed that EHD1-4 directly associate with Ankyrin, providing information on the expression and localization of these molecules in primary cardiomyocytes and demonstrating that EHD1-4 are coexpressed with ankyrin-B in the myocyte perinuclear region. Significant modulation of EHD expression follows myocardial infarction, suggesting that EHDs may play a key role in regulating membrane excitability in normal and diseased hearts (Gudmundsson et al., 2010). Retrograde transport is important for many cellular functions, including lysosome biogenesis where Vps35, a subunit of retromer, interacts with the cytosolic domain of the cation-independent mannose 6-phosphate receptor to mediate sorting of lysosomal hydrolase precursors to endosomes (Arighi et al., 2004). Annexin A2, another protein involved in acidic organelle Ca^2+^ binding and the endocytic pathway, is capable of active Ca^2+^-dependent plasma membrane resealing in vascular endothelial cells (Koerdt and Gerke, 2017), interacts with the lysosomal *N*-ethylmaleimide-sensitive factor attachment protein receptor (SNARE) VAMP8, and facilitates binding of VAMP8 to the autophagosomal SNARE syntaxin 17 to modulate the fusion of auto-phagosomes with lysosomes (Bustos et al., 2017).

Dysferlin (DYSF) acts as a Ca^2+^ sensor in Ca^2+^-triggered synaptic vesicle-plasma membrane fusion in myocytes (Rubi et al., 2015). Mutations in DYSF cause limb-girdle muscular dystrophy type 2B (LGMD2B) due to defective Ca^2+^-dependent, vesicle-mediated membrane repair (Liu et al., 1998). Loss of DYSF causes death of cardiomyocytes, notably in aging hearts, leading to dilated cardiomyopathy and heart failure in patients with LGMD2B (Wei et al., 2015). These observations in conjunction with our data demonstrate the need for further studies related to the role of DYSF in cardiac atrial pathology.

Annexin 6, involved in Ca^2+^ binding to cellular membranes such as those of acidic organelles including late endosomes (de Diego et al., 2002), were identified from our proteomic studies. As a regulator of the apical membrane events of the placenta, Annexin 6 binds in both a Ca^2+^-dependent and in a Ca^2+^-independent fashion (Riquelme et al., 2004). Annexin 6 is also involved in the trafficking events between endocytic compartments and lysosomes leading to degradation of low-density lipoproteins (LDLs) (Pons et al., 2001).

## Discussion

In this manuscript, we present a modified density gradient method of EL organelle isolation suitable for use with frozen tissue samples of at least 100mg. After confirming the identity and purity of these fractions using enzyme activity assays (Figure 2A) and Western blots (Figures 2B and S2), we performed label-free liquid chromatography with tandem mass spectrometry (LC-MS/MS) peptide analysis and present a comprehensive data set of *C. porcellus* EL focussed organelle proteomics.

The importance of lysosomes for cardiac protein turnover and the role that changes in these organelles have to this function has been known for several decades (Wildenthal and Decker, 1980). In spite of this, lysosomes have remained relatively understudied in terms of a possible role in cardiac pathogenesis. Several lysosomal storage diseases may present with cardiac abnormalities as part of disease progression, e.g. hypertrophy and conduction dysfunction in Anderson-Fabry's disease (Nair et al., 2019; Linhart and Elliott, 2007). More recently, a role for lysosomes and endolysosomes as acute signaling organelles has been identified in a number of cell types (Zhu et al., 2010, Burton and Terrar, 2021) including both atrial (Collins et al., 2011; Capel et al., 2015) and ventricular (Capel et al., 2015; Aston et al., 2017; Macgregor et al., 2007) cardiomyocytes. These observations spark a renewed interest in the function and protein composition of lysosomes and endolysosomes in this tissue. However, proteomic databases of cardiac lysosomes have not been published to this point.

The involvement of lysosomes in cardiovascular disease has been of interest for decades. Early observations presented in patients undergoing open-heart surgery suggested that the number of lysosomes in the right atrium is increased in patients with atrial septal defects (Wheat, 1964, 1965; Kottmeier and Wheat, 1967). Following these observations, in 1967, Kottmeier and Wheat (Kottmeier and Wheat, 1967) pursued experiments to see if similar findings could be produced in an experimental model. They found that the number of lysosomes increased significantly following the creation of atrial septal defects in dogs, with the most marked increase occurring in the right ventricle providing early evidence to support the role of the lysosome as an important intracellular organelle which is related to cellular stress.

Cardiomyocytes are responsible for the beat of the heart and make up the bulk of cardiac tissue by volume (Tang et al., 2009). These cells are structurally specialized for excitation-contraction coupling, containing large numbers of contractile filaments and mitochondria by volume. Although cardiomyocytes dominate heart tissue volume, non-myocytes (eg. fibroblasts, endothelial cells, vascular smooth muscle) are greater by nuclear number (Kohl and Camelliti, 2007; Pinto et al., 2016). Consequently, whole tissue analysis of the cardiac proteome is dominated by contractile, mitochondrial, and cell/ECM structural proteins. For instance, Doll et al. (2017) found 25% of identified protein molecules were from just six proteins in human heart tissue samples, of which two were contractile and two structural (Doll et al., 2017). Robust conclusions regarding the effects of physiology and disease on the proteome of other organelles therefore requires accurate enrichment of the organelle of interest. An elegant method to purify endolysosomes from cultured HeLa cells utilizing superparamagnetic iron oxide nanoparticles was published in 2017 (Tharkeshwar et al., 2017). A similar approach utilizing the uptake of latex beads was shown to allow endosome purification in cultured macrophages (Lamberti et al., 2015). Both of these methods, however, rely upon the uptake of particles to live cells, limiting their utility for analysis of frozen samples, such as might be available from large-animal and/or patient biopsies for clinical cardiac projects. The LOPIT method (Dunkley et al., 2004) on the other hand requires proteomic runs of a large number of cellular fractions, making it prohibitively expensive for smaller research groups. Instead, we focussed on improving the specificity and purity of samples produced by density-based fractionation.

Lysosomes and endolysosomes at different stages of their maturation pathway show a wide variety of densities which overlap markedly with other cellular organelles (Graham, 2001; Hibbs et al., 1965; Wolff and Pertoft, 1972; Peters and De Duve, 1974). By allowing the densest lysosomes to be collected with the Mito fraction, we have been able to separate a highly purified EL fraction at a density of 1.04 g/mL containing over 1,200 identifiable proteins by LC-MS/MS analysis (see supplemental information and PRIDE database entry PRIDE: PXD021277). Isolation of this fraction from three separate frozen *C. porcellus* atrial tissue preparations clearly established the technical reproducibility of our method, as indicated by correlation and principal component analyses (Figures 2C and 2F). Figure 2A shows the average (±SD) total enzymatic activity of both for β-galactosidase and β-hexosaminidase when compared with either TL or mitochondrial fractions (Figure 2A in each fraction). Although there was only about 10% of each enzyme activity in the EL fraction, contamination from either mitochondria or SR (which can also be found in a range of densities after tissue homogenization) (Graham, 2001) was minimal and confirmed by Western blot analysis, showing that the EL fraction was devoid of COX IV and phospholamban staining, respectively (Figures 2B and S2). The mitochondrial fraction, which contains mature dense lysosomes and the denser endolysosomes, retained 56% of the total enzyme activity. Assay of the pooled remaining gradient fractions contained less than 0.5% of the total activity. We recovered only 67% of the total activity of each enzyme which was likely to be because of the very low protein concentration in the EL fractions (~100μg/ml) (see Data S3). Quantitative proteomic comparisons of TL and EL from three cardiac atrial *C. porcellus* fractionations demonstrated enrichment of endosomal and endocytic trafficking pathways at the expense of mitochondrial proteins such as components of the electron transport chain. In particular, we identified enrichment of a range of known lysosomal markers within the EL fraction: β-galactosidase, β-hexosaminidase, β-glucosidase, Rab7, and LAMP2.

Our EL proteomic data identified the presence of multiple protein hits relevant to diseases that have been linked to dysfunctional lysosomal enzymes. These include lysosomal α-glucosidase (GAA), a key lysosomal enzyme involved in the degradation of glycogen in lysosomes (Hermans et al., 1991), the lysosomal protective protein/cathepsin A/H0VMB1, which serves a protective function by regulating stability and activity of beta-galactosidase and neuraminidase enzymes (Galjart et al., 1991) and also plays a role in galactosialidosis (Zhou et al., 1996), Clusterin/H0VVP2, identified as a potential biomarker for the LSD mucopolysaccharidosis (Skrinjar et al., 2018), and Decorin, a protein that when dysregulated contributes to cardiac fibrosis or fibrotic stiffness (Heras-Bautista et al., 2019). Glycogen phosphorylase, brain form, is a lysosomal enzyme identified in our EL fraction that regulates glycogen mobilization (Mathieu et al., 2016) and plays a prominent role as the only marker protein elevated in the early-stage of asymptomatic patients with Fabry disease (Doykov et al., 2020). In addition, we identified a major complement of lysosomal cathepsins in our proteomic data that have been previously linked with cardiovascular diseases, including cathepsins B, C, D, and Z (Guo et al., 2002; Moheimani et al., 2012; Wolinsky et al., 1978; Letronne et al., 2016; Wu et al., 2017; Thottath et al., 2019; Campden and Zhang, 2019). The successful identification of such disease-related proteins highlights the potential for use of these techniques in understanding the role of lysosomal proteins in pathology.

Our EL preparations demonstrated the enrichment of lysosomal markers β-galactosidase, β-hexosaminidase, Rab7A, and LAMP2. Apart from general lysosomal proteins, EL fractions highlighted cardiac-specific lysosomal proteins such as putative phospholipase B-like 2 (Jensen et al., 2007), whereas TL fractions highlighted cardiac-specific muscle troponin (TNNT2). Increased plasma troponin level is a risk stratification factor in AF for myocyte injury and also in myocardial infarction (Hijazi et al., 2017; Sato et al., 2004). Studies have shown that patients with AF have higher atrial natriuretic peptide (ANP) levels compared with patients in sinus rhythm, and elevated ANP levels have been shown to predict the development of paroxysmal AF in patients with congestive heart failure (Yamada et al., 2000) or following cardiac surgery (Yilmaz et al., 2006). We identified ANP in both our TL and EL fractions.

Detection of proteins specific to the atria, such as natriuretic peptides A (NPPA/ANP), highlights the utility of such methods to study atrial cardiovascular diseases. The *NPPA gene* is expressed primarily in the heart, where the expression level is higher in atria than ventricles (Potter et al., 2006).

An important protein we detect in the EL fraction is Rab7, a small GTPase that belongs to the Rab family, known to control transport to late endocytic compartments such as late endosomes and lysosomes (Guerra and Bucci, 2016). Rab7 promotes lysosomal biosynthesis and maintains lysosomal function (Distefano et al., 2015). Rab7 is directly or indirectly involved in each event that occurs between early endosomes and lysosomes. Endolysosomes are known to serve as intracellular iron storage organelles (Ward et al., 2014). Fernández et al. (2016) show that increasing intracellular iron causes EL alterations associated with impaired autophagic clearance, increased cytosolic oxidative stress, and increased cell death and these effects are subject to NAADP. Cell death triggered by altered intralysosomal iron handling is abrogated by inhibiting RAB7A activity. Alterations in the activity of Rab7 may be associated with cardiovascular diseases, lipid storage disorders, and neurodegenerative diseases (Cataldo et al., 2008; Zhang et al., 2009; He et al., 2019).

As mentioned in the introduction, previously studied lysosomal protein profiles such as Schröder et al., (2010) used liver cells for the lysosome isolations. The use of liver cells for lysosome isolation was to alter the density of lysosomes. These studies utilized injection of Triton to the animal models, which is metabolized by the liver lysosomes. Dextran accumulated hepatic lysosomes become enlarged and denser, so that both sedimentation coefficient and equilibrium density are increased in a sucrose gradient (De Duve and Wattiaux, 1966). In our endolysosome isolation protocol, such modifications or alterations were not used to manipulate the nature of the endolysosomes.

The fractionation method presented here is able to isolate cardiac lyso/endolysosomes in a robust and repeatable manner. We chose *C. porcellus* for this work due to its known electrophysiological similarities to human cardiomyocytes and long-established use for physiology data collection within the field (Terrar et al., 2007). Given the interest in lysosomes and endolysosomes as catabolic, storage and acute signaling compartments in cardiomyocytes, the use of this method for analysis of further species and in order to compare how the pathophysiology of clinical samples and disease models affects these organelles is of great interest for future research (Lübke et al., 2009; Sleat et al., 2008). Knowledge obtained from a combination of experiments performed at many levels such as genes, proteins, single cells, *in vitro* tissue engineering, isolated cardiac tissue, whole organ, and *in vivo* cardiac studies, rather than a single model or experimental technique, will lead to improved strategies for diagnosis and treatment.

### Limitations of the study

The modified density gradient protocol described in this study has the potential to identify lysosomal proteins using relatively small sample volumes. However, it is important to recognize that the proteins identified using this technique are likely to be an underestimate of the total proteins present within samples. For example, our analysis did not detect TPC1 or TPC2 proteins, EL ion channels that would be expected to be present (Wang et al., 2012; Cang et al., 2013; Grimm et al., 2014, 2017). Detecting these proteins in proteomic screens is problematic primarily due to their hydrophobicity, low levels of expression and lack of trypsin cleavage sites in their transmembrane segment sites (Vit and Petrak, 2017). We did however observe β1-integrin, which has a functional role related to TPCs (Nguyen et al., 2017), in our EL fractions. Disrupted TPC function also halts trafficking of β1-integrin, leading to accumulation in EEA1-positive early endosomes (Nguyen et al., 2017). More recently, the contribution of TPC2 and NAADP to acute and chronic β-adrenoceptor signaling in the heart has been demonstrated (Capel et al., 2015). In addition, it is not possible to rule out the possibility that some small contamination of the EL fraction with early endosomal proteins may occur during the fractionation phase. For example, besides EL proteins, we discovered potential minor contamination from early endosomal proteins in our EL fraction such as EEA1, Rab1A, and Rab6A.

## STAR★Methods

### Key resources table

**Table:** 

REAGENT or RESOURCE	SOURCE	IDENTIFIER
**Antibodies**		

Anti-LAMP2	ThermoFisher Scientific	Cat# PA1-655; RRID: AB_2134625
Anti-COX IV	Abcam	Cat# ab16056; RRID: AB_443304
Anti-Phospholamban	Abcam	Cat# ab85146; RRID: AB_10974942
PO448 Polyclonal Goat Anti-Rabbit Immunoglobulins/HRP	Agilent Dako	Cat# P044801-2; RRID: AB_2617138

**Chemicals, peptides, and recombinant proteins**		

Westar Supernova detection substrate	Cyanogen	XLS3,0020
Bolt™ 4 to 12%, Bis-Tris, 1.0 mm, Mini Protein Gel, 10-well	ThermoFisher Scientific	NW04120BOX
Lysosome Isolation Buffer	BioVision	K235-50-1
Lysosome Enrichment Buffer	BioVision	K235-50-2
Protease Inhibitor Cocktail	BioVision	K235-50-4
Sucrose	Fisher Scientific	15503022
Percoll	Santa Cruz Biotechnology	sc-500790
4-Methylumbelliferyl N-acetyl-β-D-glucosaminide	Merck (Sigma-Aldrich)	CAS 37067-30-4
Sodium Acetate Buffer	Merck (Sigma-Aldrich)	CAS 126-96-5
Triton X-100 solution	Merck (Sigma-Aldrich)	CAS 9002-93-1
Na_2_CO_3_	Merck (Sigma-Aldrich)	CAS 497-19-8
Dithiothreitol	Merck (Sigma-Aldrich)	CAS 3483-12-3
Iodoacetamide	Merck (Sigma-Aldrich)	CAS 144-48-9
Urea	Merck (Sigma-Aldrich)	CAS 57-13-6
TRIS HCL	Merck (Sigma-Aldrich)	CAS 1185-53-1

**Deposited data**		

Mass spectrometry proteomics data, Proteome Xchange via PRIDE partner repository, dataset identifier PRIDE: PXD021277	This paper	http://proteomecentral.proteomexchange.org/cgi/GetDataset?ID=PXD021277
Proteomic map of the human heart	Doll et al. 2017	https://www.nature.com/articles/s41467-017-01747-2#additional-information

**Experimental models: Organisms/strains**		

Dunkin Hartley Guinea Pig (Male) HsdDhl:DH	Envigo	Order code: 459

**Software and algorithms**		

MaxQuant software platform (version 1.6.2.3)	Cox and Mann 2008	https://www.maxquant.org
Perseus software platform (version 1.5.2.4)	Tyanova et al. 2016	http://coxdocs.org/doku.php?id=perseus:start
Prism v8	GraphPad	https://www.graphpad.com
UniProt Retrieve/ID Mapping	Uniprot KB	https://www.uniprot.org/uploadlists/
Cytoscape (Version 3.7.2)	Shannon et al. 2003	https://cytoscape.org
ClusterMaker v2.8.2	Morris et al. 2011	http://www.cgl.ucsf.edu/cytoscape/cluster/clusterMaker.shtml
Panther Gene Ontology Pathway Analysis Software	Mi et al., 2021	http://pantherdb.org

**Other**		

Thermo Scientific™ EASY-Spray™ HPLC Columns	ThermoFisher Scientific	ES803
PepMAP C18, 75 μm x 500mm, 2 μm particle HPLC column	ThermoFisher Scientific	164942
Sola HRP SPE cartridges	ThermoFisher Scientific	60109-001

### Resource availability

#### Lead contact

• Further information and requests for resources and reagents should be directed to and will be fulfilled by the lead contact, Dr Rebecca AB Burton (rebecca.burton@pharm.ox.ac.uk).

#### Materials availability

• This study did not generate new unique reagents.

### Experimental model and subject details

#### Animals

All experiments were performed in accordance with Home Office Guidance on the Animals (Scientific Procedures) Act 1986 (UK). Hearts were swiftly isolated from six healthy adult Duncan Hartley male guinea pigs (350-400g, Envigo, UK), following cervical dislocation and immediately perfused with ice-cold heparinised phosphate buffered saline (PBS). Both left and right atria were dissected, snap frozen in liquid nitrogen and stored at −80°C until required. All animals were purchased from Envigo, UK. All experimental protocols (Schedule 1) were approved by the University of Oxford, Procedures Establishment License (PEL) Number XEC303F12.

### Method details

#### Tissue homogenization

Frozen atrial tissue biopsies (100mg) were thoroughly cleaned in PBS and weighed. A minimum of 100 mg tissue is required in order to perform proteomics and biochemistry (enzyme assay and Western Blots). Each atrium was quartered and gently homogenized using a 7 mL Dounce homogenizer in Lysosome isolation buffer (LIB) [Containing 1:500 protease inhibitor cocktail (PIC) and phosphatase inhibitor (PHI) (Bio vision), (PhosSTOP Roche)]. Preparations were further homogenized in 1 mL Dounce homogenizer and transferred to chilled 1.5 mL ultracentrifugation tubes (Beckmann coulter). Sample preparations were mixed at a ratio of 1:1.5 Lysosome enrichment buffer [(LEB) (Biovision, containing 1:500 PIC)] to homogenate by inverting tubes, and were stored on ice for 5 min until the centrifugation.

#### Isolation of acidic organelles by fractionation

Samples were centrifuged at 13,000 g for 2 min at 4°C (TLX Beckmann Coulter Ultra Centrifuge) and the resulting supernatant, corresponding to the TL was collected. Further fractionation was processed using 75% of the collected TL. 1.5 mL ultracentrifuge tubes were underlaid with 750 μL of 2.5 M sucrose (Fisher Scientific) followed by 250 μL of Percoll (Santa Cruz Biotechnology). 200 μL TL was layered on top of the percoll layer and centrifuged at 27,000 g × 50 min at 10°C. The supernatant layer just above the turbid white, mitochondrial fraction (Mito fraction) was carefully removed, and the Mito fraction itself was collected separately. The collected supernatant was retained and repeated for a further centrifugation step at 29,000 g × 30 min at 15°C (500 μL of underlaid 2.5 M sucrose with overlaid 500 μL Percoll). The supernatant above the sucrose and Percoll intermediate was collected for further fractionation. Firstly, ultracentrifuge tubes were underlaid with 2.5 M sucrose and overlaid with a series of Percoll dilutions (1.11 g/mL – 1.04 g/mL in ddH_2_O). The ultracentrifuge tubes were centrifuged at 67,000 g × 30min at 4°C. The fraction at 1.04 g/mL was collected and labeled as the endolysosomal fraction (EL). N = 3 guinea pigs were used for Western Blots and proteomic analysis. A separate n = 3 biological triplicate was used for the lysozyme enzymatic analysis. The reproducibility of the fractions produced using biological triplicates can be found in Figure 2.

#### Lysosomal hydrolase activity assays

Lysosomal hydrolase activities were performed in EL, Mito fractions and TL. To fluorometrically measure the lysosome enzyme levels, artificial sugar substrates containing the fluorophore 4-methylumbelliferone (4-MU) were used. For measuring β-hexosaminidase activity, 3 mM 4-MU N-acetyl-β-D-glucosaminide (Sigma Aldrich) in 200 mM sodium citrate buffer, pH 4.5 and 0.1% Triton X-100 was used as substrate. For β-galactosidase activity, 1 mM 4-MU β-D-galactopyranoside (Sigma Aldrich) in 200 mM sodium acetate buffer, pH 4.3, 100 mM NaCl, and 0.1% Triton X-100 was used as substrate. The reaction was stopped by adding chilled 0.5 M Na_2_CO_3_, and the released fluorescent 4-MU was measured in a Clariostar OPTIMA plate reader (BMG Labtech, Ortenberg, Germany) with an excitation at 360 nm and emission at 460 nm. A standard curve for free 4-MU was used to calculate the enzyme activity. Results were calculated as total Units of enzyme activity (nmol/hr) and also normalized with respect to protein content.

##### Protein quantitation assay

Sample fractions EL or TL were mixed at a ratio of 1:1 with radio-immunoprecipitation (RIPA) buffer (Thermo scientific). Protein concentrations of all tissue fractions and TL were determined using the Bicinchoninic acid assay (BCA Protein Assay Kit, Thermo Scientific). Bovine serum albumin was used as a protein standard, and serial dilutions were prepared from the initial stock concentration of 2mg/mL to prepare a standard curve. To ensure accuracy and reproducibility, protein assays were performed in triplicate. Absorbance values were measured at 562 nm. Protein concentrations were calculated by linear regression analysis.

##### SDS/PAGE gel preparation and western blotting

Sample fractions EL and TL were solubilized, and proteins denatured using SDS/PAGE loading buffer (bio rad) and 2-mercaptoethanol (Sigma-Aldrich). Proteins were separated by gel electrophoresis (NW04120BOX, NuPAGE 4%–12% Bis-Tris protein gels, 20X MES buffer). The gel was transferred to nitrocellulose membrane (NC) (Bio-Rad) for protein transfer (X-cell-II blot module, Thermo Fisher Scientific). NC membrane was incubated in 5% skimmed milk. The primary antibodies anti LAMP2 (1:500, PA1-655, Thermo fisher scientific), anti-COX IV (1:1,000, Abcam, ab16056) and anti-Phospholamban (1:1,000, Abcam, ab85146) were incubated. Goat anti-rabbit antibody (1:2,500, Dako P0448) was used as the secondary antibody to detect the protein markers of lysosomes, mitochondria and SR, respectively. The secondary antibodies were detected via chemiluminescence using Westar Supernova (XLS3,0020, Cyanogen) and the protein bands were visualized in a ChemiDoc XRS + imager (Bio-rad with image Lab software).

##### Liquid chromatography-tandem mass spectrometry analysis

The samples were reduced by the addition of 5 μL of 200 mM dithiothreitol (30 min at room temperature) and alkylated with 20 μL of 200 mM iodoacetamide (30 min at room temperature) followed by methanol-chloroform precipitation. The pellets were resuspended in 6 M urea in 400 mM TrisHCl, pH 7.8. Urea was diluted to 1 M using 400 mM Tris-HCl, pH 7.8, and the proteins were digested with trypsin in a ratio of 1:50 (overnight at 37°C). After acidification to a final concentration of 1% formic acid, the samples were desalted on Sola HRP SPE cartridges (ThermoFisher Scientific) and dried down in a SpeedVac (3-6hours).

Samples were further desalted online (PepMAP C18, 300 μm × 5 mm, 5 μm particle, ThermoFisher Scientific) for 1 min at a flow rate of 20 μL/min and separated on an EASY-Spray column (PepMAP C18, 75 μm × 500mm, 2 μm particle, ES803, ThermoFisher Scientific) over 60 min using a gradient of 2–35% acetonitrile in 5% DMSO/0.1% formic acid at 250nL/min. Separation and analysis was performed on a Dionex Ultimate 3000 RSLC system coupled to an Orbitrap Fusion Lumos platform (both ThermoFisher Scientific) using standard parameters (Universal Method (Davis et al., 2017))*.* MS scans were acquired at a resolution of 120,000 between 400 and 1,500 *m*/*z* and an AGC target of 4.0E5. MS/MS spectra were acquired in the linear ion trap (rapid scan mode) after collision-induced dissociation (CID) fragmentation at a collision energy of 35% and an AGC target of 4.0E3 for up to 250 ms, employing a maximal duty cycle of 3 s, prioritizing the most intense ions and injecting ions for all available parallelizable time. Selected precursor masses were excluded for 30 s (Davis et al., 2017).

Mass spectrometry data were analyzed quantitatively with the MaxQuant software platform (Cox and Mann, 2008) (version 1.6.2.3), with database searches carried out against the UniProt *C. porcellus* database (UP000005447_10141). A reverse decoy database was created, and results displayed at a 1% FDR for peptide spectrum matches and protein identifications. Search parameters included: trypsin, two missed cleavages, fixed modification of cysteine carbamidomethylation and variable modifications of methionine oxidation and protein N-terminal acetylation. Label-free quantification was performed with the MaxLFQ algorithm with an LFQ minimum ratio count of 2. ‘Match between runs’ function was used with match and alignment time limits of 0.7 and 20 min, respectively. The mass spectrometry proteomics data have been deposited to the ProteomeXchange Consortium via the PRIDE partner repository with the dataset identifier PXD021277.

### Quantification and statistical analysis

#### Quantitative analysis of mass spectrometry data

Quantitative analysis of significant differences between the protein abundance of EL and TL samples and data visualization was performed using the Perseus software platform (Tyanova et al., 2016) (version 1.5.2.4) using LFQ values of biological replicates. Data for peptides where more than two values were absent from six biological replicates were excluded from the quantitative analysis and distributions of excluded values between the EL and TL fractions are represented in the Venn analysis shown in Figure S3B. Remaining data with no more than 2 missing values were uploaded as a data matrix in Perseus with the respective LFQ intensities as main columns. The data matrix was reduced by filtering based on categorical columns to remove protein groups only identified by site, reverse decoy hits and potential contaminants. A total of 2,436 proteins remained after filtering. Groups of biological replicates for EL and TL fractions were defined in categorical annotation rows. Data were log transformed (log2(x)) and normalized via median subtraction. Missing data points were imputed based on normal distribution and visualized as LFQ intensity histograms (per biological replicate) with imputed values shown separately (Figure S3A). Within the EL fraction, this corresponded to only 7% of proteins, and the inclusion of imputed values did not affect the normal distribution of data as confirmed using D'agostino & Pearson omnibus K2 test (Graphpad Prism v8). Principal component analysis (PCA) (Figure 2F) was performed on 100% valid values. A volcano plot was generated based on LFQ intensities applying two-way Student's t-test to probe for significant difference of protein abundance between EL and TL samples (Figure 3A). A permutation-based false-discovery rate (FDR) was determined with 250 randomizations and S_0_ = 0.1 (default). Out of the total 2,436 proteins, 1,254 proteins were accounted with 99% confidence level at 5% FDR. Of these 690 proteins were significantly depleted in EL compared to TL, and 564 proteins were significantly enriched in EL (Figure 3A). For comparison with previously published proteomic data for human atria (Doll et al., 2017), LFQ intensity histograms were plotted for pooled TL data from the current study and compared with LFQ intensities published by Doll et al. (2017) for human left and right atria (Figure S4).

#### Network analysis

The functional networks of the statistically enriched proteins acquired from the volcano plot were analyzed using Cytoscape (Shannon et al., 2003) (Version 3.7.2) and Panther pathway analysis software (Mi et al., 2021) (Figure 3B). *C. porcellus* proteins were converted to *H. Sapiens* with UniProt Retrieve/ID mapping to identify the reviewed proteins with 100%–50% similarity in the molecular function through biological and molecular pathways. The Cytoscape 3.7.2. programme was used in order to investigate associations and relations between EL proteins and protein interaction data was extracted from the STRING, KEGG and Reactome databases. The protein match to the molecular networks aligned with medium confidence of 0.4 and STRING identified a total number of 125 proteins involved in a single functional network and 7 proteins displayed in a detached cluster within the endo-lysosomal compartment. We selected the 564 highest abundant EL fraction proteins and clustered into the main network. We applied perfused force-directed layout on the network using clusterMaker 2.8.2 (Morris et al., 2011). Functional enrichment analysis was performed for the two clusters. Pearson correlation coefficient, heat maps, PCA and histograms (Figures 2 and S3A) were produced to assess the reliable correlation between the sample triplicates. The heat maps were produced using Euclidian distance and K-mean clustering. Plots were generated using Perseus software (v. 1.5.2.4) and presented for publication using Instant Clue (Nolte et al., 2018) The protein profile plots were created using Pearson clustering. The unavailability of the characterized whole guinea pig proteome limitation was minimized by selecting 100%–50% overlap of the proteins with the closest species in the phylogenetic tree. These selections were automatically identified by proteome databases such as Uniprot and proteomic tools Perseus, Cytoscape, String and Panther Pathway.

## Data Availability

• The mass spectrometry proteomics data generated in this study have been deposited to the ProteomeXchange Consortium via the PRIDE partner repository with the dataset identifier PRIDE: PXD021277 and are publicly available as of the date of publication. • This paper does not report original code. • Any additional information required to reanalyze the data reported in this paper is available from the lead contact upon request.

## Supporting citations

The following references appear in the supplemental information: Anderson et al., 2018; Austin et al., 2012; Bhattacharyya et al., 2011; Carcel-Trullols et al., 2017; Chang et al., 2014; Chen et al., 2013; Chung et al., 2019; De Seranno et al., 2006; Dera et al., 2019; Erasmus et al., 2016; Gittleman et al., 2016; Gu et al., 2017; Hallett et al., 2018; Hamdan et al., 2014; Hedberg-Oldfors et al., 2019; Helbig et al., 2019; Hoock et al., 1997; Hung et al., 2014; Kang et al., 2017; Khalid et al., 2016; Kook et al., 2016; Kostelansky et al., 2006; Levi and Finazzi, 2014; Li et al., 2020; Macri et al., 2015; Martinez and Goud, 1998; Mizunoe et al., 2020; Mohammadzadeh et al., 2020; Mohler et al., 2003; Määttä et al., 1994; Nguyen et al., 2019; Orenstein and Cuervo, 2010; Rezaie et al., 2002; Salabarria et al., 2020; Scheidel et al., 2018; Sjodin et al., 2019; Tan et al., 2015; Tang et al., 2019; Vaibhava et al., 2012; Wang et al., 2019a, 2019b; Ye et al., 2020; Zielonka et al., 2019.

## Supplemental information

Document S1. Figures S1–S4

Table S1. Key proteins identified from *C. porcellus* EL fraction and grouped according to protein function with information relating to their known roles in disease, related to Figure 3

Data S1. Table of enzyme activities, related to Figure 2

Data S2. TL most abundant protein list, related to Figures 2 and 3

Data S3. EL most abundant protein list, related to Figures 2 and 3

Data S4. TL proteins for panther analysis, related to Figure 3

Data S5. EL proteins for panther & cytoscape analysis, related to Figure 3

Data S6. Enrichment ShinyGo pathway analysis, related to Figure 4
