# Supplementary material for: A modified density gradient proteomic-based method to analyze endolysosomal proteins in cardiac tissue
Source: iScience. 2021 Aug 4;24(9):102949. doi: 10.1016/j.isci.2021.102949 (PMC8384914; doi:10.1016/j.isci.2021.102949)
Supplement: Document S1. Figures S1–S4 [file mmc1.pdf]

iScience, Volume *24*

Supplemental information

A modified density gradient proteomic-based method to analyze endolysosomal proteins in cardiac tissue

Thamali Ayagama, Samuel J. Bose, Rebecca A. Capel, David A. Priestman, Georgina Berridge, Roman Fischer, Antony Galione, Frances M. Platt, Holger Kramer, and Rebecca A.B. Burton

Supplementary Figures

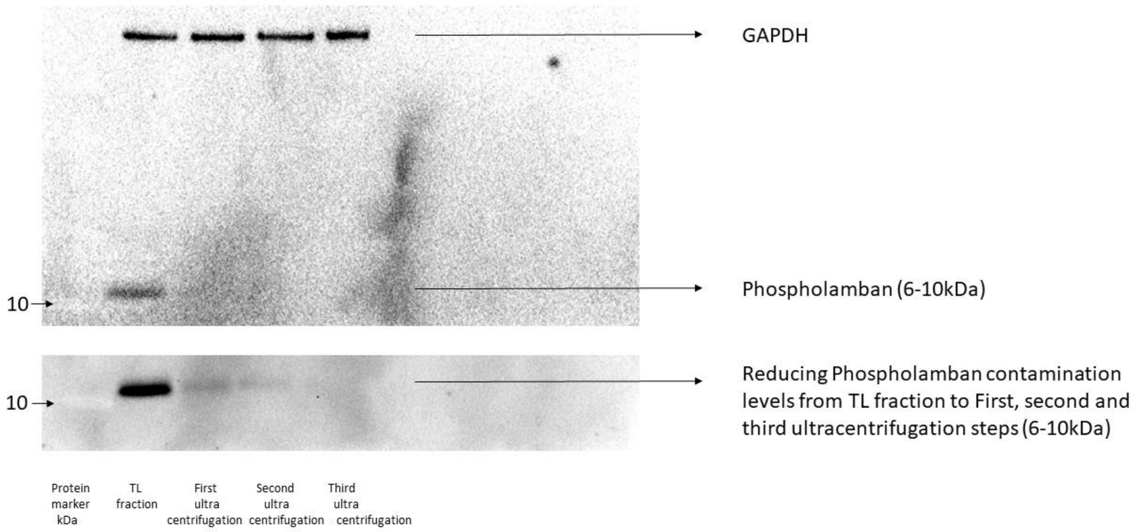

Supplementary Figure 1. Confirmation of purity of EL enriched fractions, related to Figures 1 and 2. Western blot demonstrates increasing purity of EL enriched fractions as shown by reduced levels of phospholamban from the TL fraction to the first, second and third ultracentrifugation steps. Bottom panel shows same blot without GAPDH antibody.

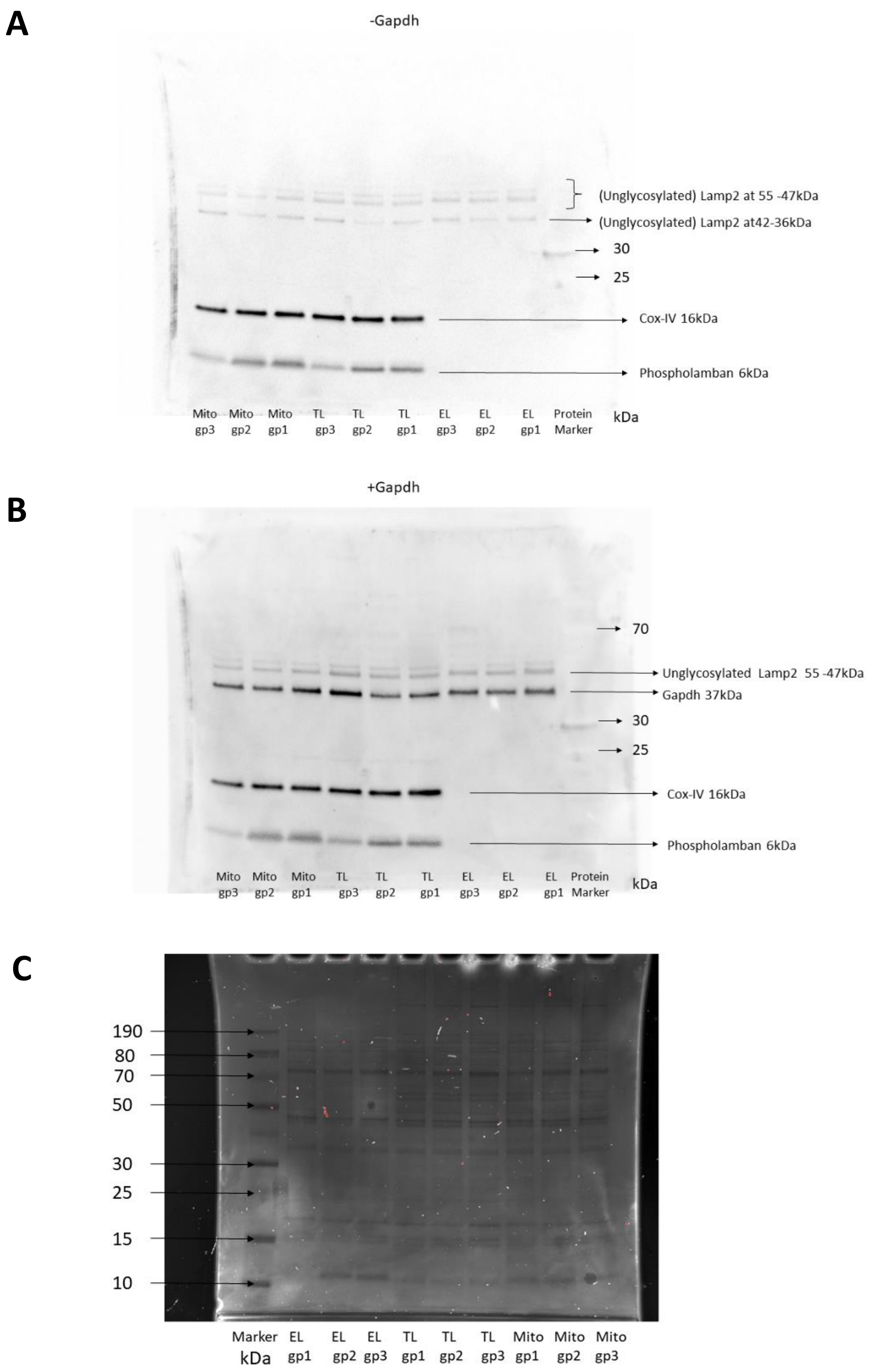

Supplementary Figure 2. Western blots for markers for lysosome (LAMP2), Mitochondria (COXIV) and SR (phospholamban), **related to Figure 2**. **A**, Complete Guinea pig (n = 3) western blot corresponding to Figure 2B. The western blots of the guinea pig atrial tissue used for lysosome assays displayed positive COXIV bands for the presence of mitochondria in Mito fraction and TL. EL fractions did not display bands for COXIV. **B**, Guinea pig (n = 3) western blots including Gapdh loading control. **C**, Coomassie stained gel demonstrating protein loading.

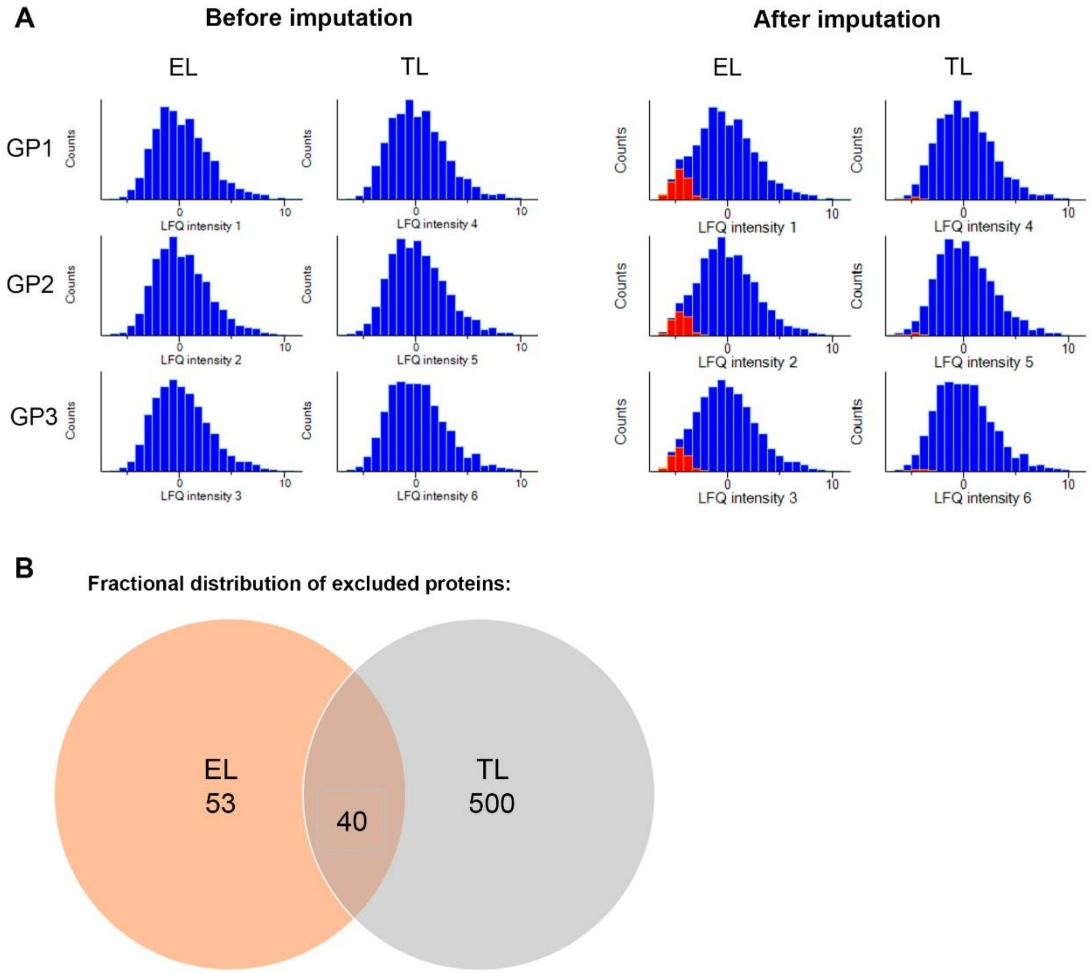

Supplementary Figure 3. Technical reproducibility of proteomic measurements and representation of quality control, related to Figures 2 and 3. A, Histograms generated before (i) and after (ii) inclusion of imputed values: Histograms are produced of the triplicated LFQ intensity datasets, demonstrating the normal distribution of protein intensities. In ii, contributions of imputed values are shown separately in red. Data was median subtracted for normalization. **B**, Venn diagram representing relative distribution between fractions for proteins with more than two missing values that were excluded from subsequent analysis; EL = 53, TL= 500 and 40 proteins were detected in both EL+TL.

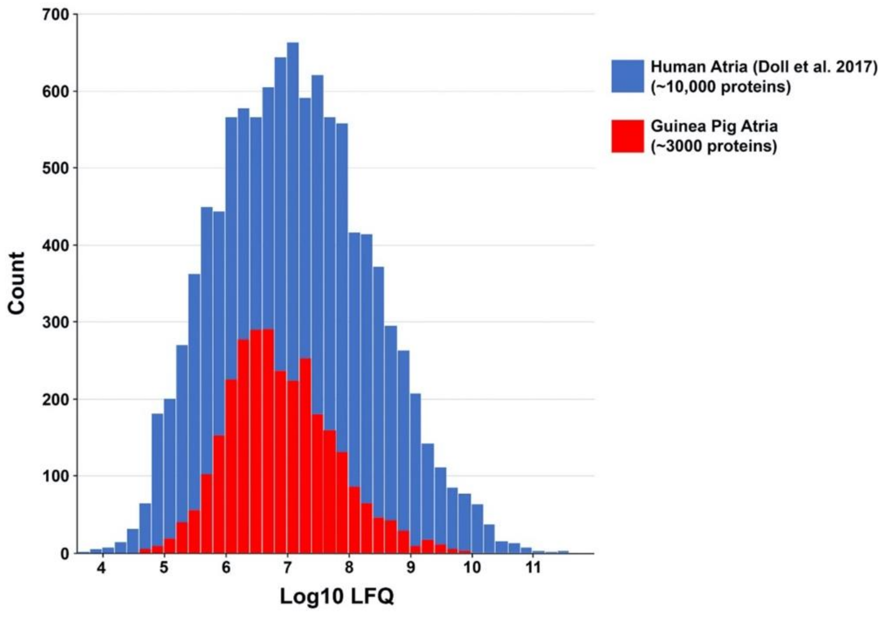

Supplementary Figure 4. Comparison of proteomic data between human and guinea pig atria, related to Figures 2 and 3. Histograms show the total proteins identified in the TL from our study (red) compared to proteins identified from human left and right atrial tissue samples (blue) in the study by Doll *et al.* (Doll *et al.*, 2017).
